# Genomic approaches for understanding dengue: insights from the virus, vector, and host

**DOI:** 10.1186/s13059-016-0907-2

**Published:** 2016-03-02

**Authors:** Shuzhen Sim, Martin L. Hibberd

**Affiliations:** Infectious Diseases, Genome Institute of Singapore, Singapore, 138672 Singapore; Faculty of Infectious and Tropical Diseases, London School of Hygiene and Tropical Medicine, London, WC1E 7HT UK

## Abstract

The incidence and geographic range of dengue have increased dramatically in recent decades. Climate change, rapid urbanization and increased global travel have facilitated the spread of both efficient mosquito vectors and the four dengue virus serotypes between population centers. At the same time, significant advances in genomics approaches have provided insights into host–pathogen interactions, immunogenetics, and viral evolution in both humans and mosquitoes. Here, we review these advances and the innovative treatment and control strategies that they are inspiring.

## Background

**Fig. 1:**
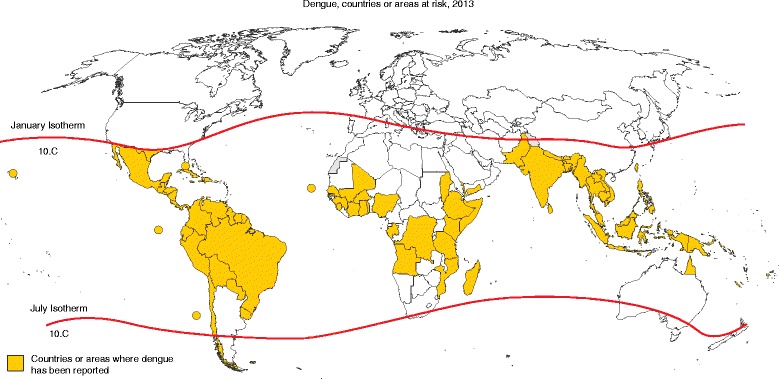
Countries or areas where dengue has been reported, 2013. Image taken from [199]; data on dengue are from the WHO

Although only nine countries had experienced severe dengue epidemics prior to 1970, the disease is now endemic in more than 100 countries (Fig. 1) [1]. Today, an estimated 3.6 billion people live in areas at risk for epidemic transmission, with nearly 400 million infections occurring annually [2]. This significant public health threat is no longer confined to the tropics — autochthonous dengue transmission has now been recorded in several European countries [3], and in 2014, Japan reported its first outbreak of the disease in 70 years [4].

Dengue virus (DENV) is a positive-sense, single-stranded RNA virus of the family *Flaviviridae*. The four DENV serotypes (DENV1, 2, 3, and 4) are primarily transmitted between humans by the mosquito *Aedes aegypti*, with *Aedes albopictus* as a secondary vector. In many cities, rapid urbanization has resulted in densely packed human and mosquito populations and in an increased availability of mosquito breeding sites, creating ideal conditions for transmission. At the same time, increased global travel efficiently transports viruses and vectors between population centers [5]. In the future, climate change and consequent increases in temperature and humidity are largely expected to further increase the risk of dengue transmission in both tropical and temperate regions [6–8].

The neglected tropical diseases (NTDs) disproportionately affect the world's poorest populations, and are caused by a diverse array of viral, bacterial, protozoan, and helminthic pathogens. Collectively, they have an approximate global prevalence of 1.0–1.2 billion cases [9, 10]. Dengue, one of the best-studied NTDs, is among the 17 prioritized by the World Health Organization (WHO) [9]. Funding for dengue research has increased steadily over the past decade, with the vast majority of resources going toward vaccine development [11]. Despite this, an effective tetravalent (eliciting immunity against all four serotypes) vaccine remains elusive [12–15], as does an antiviral that is effective against the virus. Research gaps also exist in the areas of vector control and surveillance.

Technologies that allow us to examine complex host–pathogen interactions from a whole-genome perspective have become more widely available and affordable. This progress is crucial for the development of treatment and control strategies for NTDs, which often suffer from a lack of basic research. This review concentrates on whole-genome studies that have been undertaken on DENV, mosquitoes, and humans to address key research questions. From the virus perspective, we explore studies of inter- and intra-host genetic variation and its effect on viral fitness and transmission. From the host perspective, we review gene-expression-profiling analyses characterizing mosquito and human responses to DENV infection, as well as studies identifying genes that affect susceptibility to virus infection. We further explore the impact of the mosquito midgut microbiota on the virus. Finally, we review the role that genomics studies can play in informing and enabling clinical management, vaccine and drug development, and vector-control strategies.

## Virus genetic diversity and its implications for evolution and virulence

### Viral inter-host variation

In humans, infection with DENV results in a spectrum of clinical outcomes, ranging from self-limiting, uncomplicated dengue fever (DF) to the more severe dengue hemorrhagic fever (DHF), which is characterized by increased vascular permeability and thrombocytopenia (low platelet count). In the most severe cases, these hemorrhagic manifestations lead to potentially fatal hypovolemic shock, a condition known as dengue shock syndrome (DSS). Infection with one serotype of DENV confers short-lived immunity against heterologous serotypes, but after this immunity wanes, heterologous infection carries an increased risk of severe disease. This phenomenon, known as enhancement, may be mediated through antibody responses that are directed against the previous instead of the current serotype, leading to increased viral replication [16] (reviewed in [17]).

**Fig. 2:**
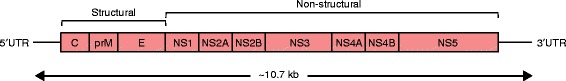
The dengue virus genome. The ~10.7 kb DENV genome encodes three structural proteins (capsid [*C*], premembrane [*prM*], and envelope [*E*]) and seven non-structural (*NS*) proteins (NS1, NS2A, NS2B, NS3, NS4A, NS4B, and NS5). *UTR* untranslated region

The ~10.7 kb DENV genome encodes three structural proteins (capsid [C], premembrane [prM], and envelope [E]) and seven non-structural (NS) proteins (NS1, NS2A, NS2B, NS3, NS4A, NS4B, and NS5) (Fig. 2) [18, 19]. Within each of the four DENV serotypes, which share ~65–70 % amino acid sequence similarity [20], virus strains are further classified into distinct genotypes, which can vary by ~6–8 % at the nucleotide level and by 3 % at the amino acid level [21–24]. Amplification and sequencing of DENV directly from patient samples has now become routine in many laboratories, making a large number of DENV sequences available for studies on genetic variation and viral evolution.

Owing to its association with frequent and severe epidemics, DENV2 is the serotype for which the most sequence data are available. Phylogenetic analysis of DENV2 E gene sequences reveals five genotypes, known as the American, Cosmopolitan, Asian, Southeast Asian/American, and sylvatic genotypes [25–27]. Although these genotypes are largely distinguished by their geographical distributions, they also commonly contain viruses from different locations, an indication of how far infected humans and mosquitoes can spread the virus. This pattern is also true for the other DENV serotypes [21, 28], and is likely to become more complex with increased human movement.

Epidemiological data suggest that certain viral genotypes may differ in their ability to cause severe disease (although given suitable conditions, such as enhancing levels of antibody, all serotypes and genotypes have the potential to do so). The introduction of an Asian genotype of DENV2, probably from Vietnam, into Cuba in 1981 and its subsequent spread into the Americas was followed by major outbreaks of DHF [22, 29]; later phylogenetic analyses suggested an association between imported Asian DENV2 genotype sequences and DHF/DSS [25]. American genotype viruses, by contrast, are not known to cause severe dengue, even in outbreaks where secondary infection is common, like that in Peru in the early 1990s [30].

Whole-genome comparisons revealed characteristic differences between DHF/DSS-causing Asian genotype viruses and American genotype viruses [31]. Most notably, differences at amino acid 390 of the E protein and in the 5' and 3' untranslated regions (UTRs) affect viral replication in monocyte-derived dendritic cells and in macrophages, with the introduction of the American genotype variant attenuating replication of Asian genotype viruses [32, 33]. Asian genotype viruses also displayed more efficient infection and dissemination in *Ae. aegypti* mosquitoes [34, 35], suggesting that they are also more easily transmitted.

It remains to be seen whether similar distinctions exist within the DENV1 and DENV4 serotypes [36], but certain genotypes of DENV3 have been associated with DHF outbreaks in Tahiti, Fiji, and Sri Lanka (reviewed in [19, 28]). Functional studies to identify and characterize potential virus determinants of pathogenicity, as described above for DENV2, are lacking.

Some researchers have recently proposed the emergence of a fifth serotype of DENV, based on an atypical virus isolated in 2007 from a patient in Borneo. Reported to be phylogenetically distinct and to elicit an antibody response that differs from that initiated by DENV serotypes 1–4, this virus is thought to circulate among non-human primates, but whether sustained transmission between humans can occur remains unclear [37]. A recent study suggests, however, that there is more antigenic heterogeneity within serotypes than previously thought. By mapping neutralizing antibody responses to a range of DENV1–4 isolates, Katzelnick et al. found that many viruses, while falling within a single serotype on the basis of gene sequence, were as antigenically similar to viruses of other serotypes as they were to each other [38]. This finding has important implications for vaccine development, and claims of 'atypical' virus isolates should also be evaluated with it in mind.

Although we have focused on viral genetics in this section, disease outcomes are in fact influenced by complex interactions between viral and host immunological factors. This was demonstrated in a study carried out in Nicaragua, where an abrupt increase in severe disease has been observed over several years of DENV2 epidemics. OhAinle et al. [39] found that severe disease in later epidemics was associated with waning DENV1 immunity in the population, as well as with replacement of the circulating DENV2 NI-1 virus clade with a new and fitter clade, NI-2B. The contribution of virus clade to increased severity was best explained in the context of serotype-specific immunity — NI-1 viruses were more virulent in children who were immune to DENV1, while NI-2B viruses were more virulent in DENV3-immune children [39].

### Viral intra-host variation in humans

Like other RNA viruses, DENV has a RNA-dependent RNA polymerase (RdRp, encoded by NS5) that has an intrinsically high error rate (~1 × 10^-4^, corresponding to approximately one mutation per 11 kb DENV genome) [40]. When coupled with a burst size of ~10^3^–10^4^ genomes per cell [32, 41], this error rate results in a population of related but genetically distinct viral genomes, organized around a consensus sequence, within each individual human or mosquito host. Sometimes termed a quasispecies, these variants are thought to interact cooperatively on a functional level, and to contribute collectively to the overall fitness of the virus population, allowing it to adapt to changing environments (reviewed in [40]). High-fidelity poliovirus mutants are markedly attenuated and less able to access the central nervous system in mouse models [42, 43]; high-fidelity mutants of chikungunya virus (a mosquito-borne alphavirus) also show reduced replication and dissemination in both mice and *Ae. aegypti* [44], demonstrating the importance of genetic diversity during infection. For mosquito-borne viruses, intra-host genetic diversity may also offer an advantage for surviving the distinct selection pressures encountered when cycling between human and mosquito hosts [44, 45].

Until recently, studies of DENV intra-host genetic diversity in patient samples involved the Sanger-sequencing of multiple clones of short regions of one or two viral genes, such as C, E, and NS2B [23, 46–49]. These studies confirmed the presence of measurable intra-host genetic variation in DENV populations, with diversity levels and variant positions differing widely among patients. One study reported lower viral intra-host variation in DHF/DSS than in DF patients, suggesting a relationship between genetic diversity and clinical outcome [47]. Another study found no such association between intra-host variation and disease severity, viremia levels, or immune status [23]. These contrasting results may be attributable to a number of variables, including differing immune status and host genetics of patient populations, different circulating virus strains, differences in variant-calling approaches, and statistical noise from low sampling rates. Notably, the latter study, by Thai *et al*. [23], used a statistically rigorous approach to differentiate true variants from those arising from PCR or sequencing errors, and this probably resulted in their finding levels of diversity that were much lower than those reported previously. Despite this, multiple distinct lineages of the same DENV1 genotype were detected within individual patients, suggesting that mixed infections may be an important contributor to intra-host genetic diversity [23].

High-throughput next-generation sequencing (such as that on the Illumina and 454 platforms) is now being used to study intra-host genetic variation across the entire DENV genome at high coverage (and thus increased sampling rates) [50–53]. This has uncovered varying intra-host diversity levels among both viral genes and different regions of the same gene, indicating that selection pressures vary across the genome. For example, a study of Nicaraguan DENV2 patient samples found that highly immunogenic E-protein domains displayed high levels of intra-host genetic diversity, suggesting that immune selection pressures on viral variants operate even during acute infection [50]. The viruses in this study were classified into two clades, separated by nine amino acid differences, within the same genotype. Intra-host diversity levels were found to be strongly associated with clade identity, suggesting that some amino acid differences may impact diversity, with those in NS5, for example, having the potential to affect polymerase error rates.

### Viral intra-host variation in mosquitoes

In mosquitoes, RNA interference (RNAi), a key antiviral defense mechanism in insects, has been proposed to be a driver of viral intra-host genetic diversity. This has been best studied in the *Culex* mosquito*–*West Nile virus (family *Flaviviridae*) system [54], in which greater intra-host diversity levels were reported in mosquitoes than in vertebrate hosts [55, 56]. At the same time, host alternation subjects arboviruses to frequent and significant drops in population size. Only a small percentage of the total virus population circulating in the human is ingested by the mosquito host in its ~2 μl bloodmeal, and an even smaller number of viruses will eventually seed infection in the mosquito gut. Drops in population size also occur during subsequent spread through various tissues and organs of the insect, as well as during the injection of microliter volumes of infected saliva into human hosts. It is unclear how these processes shape the diversity and repertoire of the viral population.

To track changes in viral intra-host genetic diversity during human-to-mosquito transmission, we and collaborators infected *Ae. aegypti* mosquitoes by allowing them to feed directly on DENV2-infected patients [57]. We then deep-sequenced human- and matched mosquito-derived DENV populations, and used the variant-caller LoFreq [51] to detect true single nucleotide viral variants [58]. Human-, mosquito-abdomen-, and mosquito-salivary-gland-derived DENV populations showed dramatically different variant repertoires: >90 % of variants were lost at each stage of transmission, most probably due to large population drops that occur during the seeding of infection. Overall levels of viral diversity remained unchanged, however, suggesting that a new array of variants is regenerated by the time of sampling.

The selection pressures imposed on certain viral genes also differed between human and mosquito hosts. Specifically, we observed stronger selection pressures on the prM, E, and NS1 genes in human-derived populations than in mosquito-derived populations, consistent with these gene products being known targets of the human antibody response [59], which has no insect equivalent. By contrast, most variants, even when maintained across transmission stages, appeared to be of neutral fitness value in the mosquito host as their frequencies remained largely unchanged [58].

Viral deep sequencing may also be used to identify potential drug targets. A recent study identified a shared cold-spot, or region with a statistically significant lack of variants, in the NS3 gene of DENV1 populations from human sera and from *Ae. aegypti* and *Ae. albopictus* mosquitoes that were intrathoracically inoculated with this sera. The authors suggest that such genetically constrained regions, in which drug-resistant mutations are presumably less likely to arise, can be further explored as antiviral targets. Interestingly, while variants that were common to both mosquito species were observed, there was also evidence of species-specific selection pressures, with two variants in NS5 reproducibly appearing in *Ae. aegypti* but not in *Ae. albopictus* [60].

To enable more detailed phylogenetic analyses, molecular biological and statistical methods have been developed to reconstruct full-length viral haplotypes on the basis of short-read sequence data [61, 62]. The continuously increasing length of sequence reads (such as the multi-kilobase reads now provided by the Pacific Biosciences RS platform) should facilitate such approaches, and also make it possible to obtain viral haplotypes directly from sequence data.

Despite the growing number of studies characterizing DENV intra-host genetic diversity, the impact of this diversity on viremia or clinical outcome is not well understood, and studies using rigorous variant-calling algorithms to filter out process errors have found no such associations [23, 50]. However, most studies have sampled virus populations during the acute, viremic phase of the disease; it will be important to determine if disease severity may be associated with the genetic diversity of the infecting viral population, rather than with diversity after the onset of symptoms.

## Virus interactions with the mosquito vector

### Immune responses to DENV

Once ingested in a bloodmeal taken from an infected human, DENV first infects the midgut epithelium of the mosquito. It subsequently disseminates to other organs via the hemolymph, finally infecting the salivary glands. The virus is secreted into mosquito saliva, and injected into a human host during a subsequent blood-feeding event [5]. Mosquitoes remain infected and able to transmit the virus for life (~2–3 weeks in the wild), but DENV does not appear to exert a fitness cost on the vector during natural infection [63].

The mosquito innate immune system can distinguish between broad classes of microbes, and mounts a potent response against viruses, bacteria, and fungi (reviewed in [64]). Whole-genome DNA microarray and RNA-sequencing analyses revealed that DENV infection of the mosquito midgut, carcass, and salivary gland transcriptionally regulates numerous genes related to innate immunity, metabolism, and the stress response [65–69]. Among the immunity-related genes, those associated with Toll signaling [65–67], and to a lesser extent Janus kinase/signal transducers and activators of transcription (JAK-STAT) signaling, were prominently represented [65, 68]. RNAi-mediated gene knockdowns in adult mosquitoes subsequently confirmed key roles for these two pathways in anti-DENV immunity [65, 68]: knockdown of Cactus, a negative regulator of the Toll pathway NF-kB-like transcription factor Rel1, renders mosquitoes more refractory to DENV infection; whereas knockdown of the adaptor protein MyD88, which is required for Toll signal transduction, increases viral loads in the insect [65]. Similarly, knockdown of protein inhibitor of activated STAT (PIAS), a negative regulator of the JAK-STAT pathway, reduces infection levels, whereas knockdown of the pathway receptor Dome or the JAK ortholog Hop has the opposite effect [68].

The Toll (Rel1)-regulated transcriptome, as determined by expression profiling of Cactus-silenced mosquitoes, comprises almost 2000 genes, consistent with the pathway's diverse roles in immunity and development. Immunity-related signaling molecules and effector genes feature prominently in this dataset, and overlap considerably with those regulated by DENV infection [65]. The Toll-regulated, DENV-induced antimicrobial peptides (AMPs) cecropin and defensin have been shown by gene knockdown to inhibit DENV proliferation in mosquitoes, possibly through disruption of host cell or viral envelope membranes [66, 70]. Although the Toll pathway has clear antiviral roles, more functional evidence is required to implicate other Toll-regulated genes in anti-DENV defense mechanisms.

By contrast, immunity-related genes comprise only a small proportion of the mosquito’s JAK-STAT-regulated transcriptome (as determined through expression profiling of PIAS-silenced mosquitoes), suggesting that this pathway restricts DENV through a non-classical response [68]. Two JAK-STAT-regulated, DENV-induced putative effectors that restrict DENV replication have been identified, but their modes of action remain uncharacterized. Dengue virus restriction factor 1 (DVRF1) is a putative transmembrane protein that presumably functions as a pathway receptor, and DVRF2 contains antifreeze and allergen domains and may be involved in virus recognition [68].

### RNAi defense mechanism

The RNAi mechanism is a key *Ae. aegypti* defense against DENV and other arboviruses [71–73]. The exogenous small interfering RNA (siRNA) response, the best studied of the RNAi pathways, is initiated when long, virus-derived double-stranded RNA (dsRNA) is recognized and cleaved by Dicer-2 (Dcr2) into siRNAs, usually of 21 base pairs (bp) in length. These duplex siRNAs are loaded onto the RNA-induced silencing complex (RISC), which unwinds them, degrading one of the strands and using the other for targeted degradation of single-stranded viral RNA that has a complementary sequence (reviewed in [74]).

Deep sequencing of small RNAs from DENV-infected *Ae. aegypti* revealed nearly equal ratios of positive- to negative-sense DENV-derived small RNAs, suggesting that most small RNAs are derived from dsRNA replicative intermediates rather than from intra-strand secondary structures [75]. Interestingly, only 0.005–0.06 % of all small RNA reads map specifically to DENV [75, 76], a percentage similar to that observed for West Nile virus in *Culex* mosquitoes [54] but much lower than that for alphaviruses (10 % for Sindbis virus in *Ae. aegypti*) [77]. It has been proposed that sequestration of flavivirus replication complexes in membrane-enclosed vesicles in mosquito (and mammalian) cells [78], which restricts Dcr2 access to dsRNA replicative intermediates, may account for this. Further, given the low abundance of DENV-derived small RNAs, it has also been suggested that Dcr2 cleavage of dsRNA alone is sufficient to keep viral replication in check [75].

Although 21-bp virus-derived siRNAs dominate during middle- and late-stage infection [75, 76], virus-derived small RNAs of 24–30 bp in length are the most prevalent species during early-stage infection [76]. These longer small RNAs are most likely generated by the PIWI RNA (piRNA) pathway, suggesting a role for this Dcr2-independent pathway in anti-DENV defense [76], as has been proposed for other arboviruses [79, 80].

### Genetic and transcriptomic variation underlying vector competence

Vector competence — the intrinsic ability of a mosquito to become infected by, support replication of, and transmit a pathogen — varies widely between and within mosquito populations [81–84]. It is genetically determined, but is also influenced by environmental factors (reviewed in [85]). *Ae. aegypti* vector competence for DENV appears to be an additive trait that is under the control of multiple genetic loci [86, 87]. Mapping studies have identified several quantitative trait loci (QTLs) that are associated with the ability of DENV to establish infection in the midgut (cross the midgut infection barrier) or to disseminate out of it and infect other tissues (cross the midgut escape barrier) [87–89]. The specific genes or polymorphisms involved, however, have yet to be identified definitively.

In addition, vector competence is influenced by genotype-by-genotype (GxG) interactions, in which infection and dissemination are affected by the specific combination of mosquito and virus genotypes [90, 91]. This complicates genetic mapping because the resistance loci or alleles may differ depending on the mosquito population and the virus strain [92]. For example, natural polymorphisms in *Ae. aegypti* Dcr2 have been found to be associated with resistance to DENV infection, but in a virus isolate-specific manner. It has been proposed that this specificity is due to differences in the affinity of Dcr2 for particular viral dsRNA sequences [93].

Roughly two-thirds of the ~1.4 Gb *Ae. aegypti* genome is composed of transposable elements, repeats, or duplications [94, 95], making marker development difficult. Tools are being developed to circumvent these challenges — for example, a recently published single-nucleotide polymorphism (SNP) chip is capable of screening 50,000 SNPs in 96 samples simultaneously [95] — and should facilitate more comprehensive, genome-wide studies of vector competence. Targeted-enrichment and deep-sequencing approaches have been developed for the detection of polymorphisms and copy number variations that are associated with insecticide resistance in *Ae. aegypti* [96]; these approaches could potentially also be adapted to studies of vector competence.

Variation at the transcriptome level is also associated with susceptibility to DENV [84, 97–100]. Microarray expression profiling of the DENV-responsive transcriptomes of refractory and susceptible *Ae. aegypti* strains revealed differentially expressed gene clusters. These were predominantly related to metabolism and to the stress response, as well as to a common core of DENV-responsive genes, which were mostly related to key signaling pathways, including the JAK-STAT, Wnt, mitogen-activated protein kinase (MAPK), and mammalian target of rapamycin (mTOR) pathways [97–99]. In another study, performed in the absence of DENV infection, expression profiling of a panel of strains from geographically distinct endemic regions found that numerous immunity-related transcripts were more abundant in refractory strains than in susceptible ones, suggesting that basal levels of immune activation impact susceptibility [84]. Given the well-documented role of gut bacteria in stimulating basal immunity in mosquitoes [65, 101, 102], it is possible that the co-evolution of these strains with unique suites of microbial species may have resulted in transcriptomic divergence.

Mosquito genes found (using genomic methods) to be associated with vector competence for DENV are listed in Table 1.

**Table 1:** Genes associated with susceptibility to DENV in humans and mosquitoes

Host	Gene	Type of variation	Method	Associated with	Reference(s)
Human	*MICB*	Genetic	GWAS	Severe dengue	[153]
Human	*PLCE1*	Genetic	GWAS	Severe dengue	[153]
Mosquito	Early trypsin	Genetic	QTL analysis	Midgut infection	[86, 87]
Mosquito	*Dicer-2*	Genetic	Candidate gene sequencing	GxG interactions	[93]
Mosquito	Cecropin	Transcriptomic	Microarray/RNAi	Midgut, salivary gland infection	[66, 70]
Mosquito	Defensin	Transcriptomic	Microarray/RNAi	Midgut infection	[70]
Mosquito	*DVRF1*	Transcriptomic	Microarray/RNAi	Midgut infection	[68]
Mosquito	*DVRF2*	Transcriptomic	Microarray/RNAi	Midgut infection	[68]
Mosquito	Vacuolar ATPase	Transcriptomic	Microarray/RNAi	Midgut infection	[84, 189]
Mosquito	High mobility group box protein (HMGB)	Transcriptomic	Microarray/RNAi	Midgut infection	[84]
Mosquito	ML33	Transcriptomic	Microarray/RNAi	Midgut infection	[186]
Mosquito	NPC1b	Transcriptomic	Microarray/RNAi	Midgut infection	[186]

*GWAS* genome-wide association study, *GxG* genotype-by-genotype, *QTL* quantitative trait loci, *RNAi* RNA interference

### Impact of the mosquito microbiome on vector competence

Mosquitoes harbor bacterial communities that have diverse impacts on nutrition, digestion, metabolism, development, immunity, and other aspects of insect biology [103, 104]. The adult mosquito gut, in particular, is a site of complex reciprocal interactions between the natural gut microbiota, the mosquito host response, and bloodmeal-acquired pathogens such as DENV. Importantly, the gut microbiome is known to influence vector competence for DENV and other mosquito-borne pathogens (reviewed in [105]).

Removal of native gut bacteria by antibiotic treatment has been reported to render *Ae. aegypti* more susceptible to DENV infection; these aseptic mosquitoes also display reduced levels of AMP expression [65]. In addition, several bacterial isolates derived from field-collected mosquitoes have the ability to inhibit DENV replication when reintroduced into aseptic mosquito midguts [102, 106]. In some cases, bacteria are thought to activate basal level production of immune effectors such as AMPs, and thus prime the mosquito against subsequent viral infection [65, 70, 102]. This is consistent with known functional overlaps between the mosquito antibacterial and antiviral responses [65, 66, 70, 102]. Other bacteria have been shown to inhibit DENV independently of the mosquito, and are thought to produce secondary metabolites that have direct antiviral activity [106].

Bacteria of the genus *Wolbachia* are maternally inherited, intracellular endosymbionts that naturally infect a wide range of insects, including *Drosophila* and *Ae. albopictus*, but not *Ae. aegypti*. Stable trans-infection of *Ae. aegypti* has been achieved through embryo microinjection [107, 108], producing mosquitoes that are more resistant to a range of pathogens, including DENV, chikungunya virus (CHIKV), yellow fever virus (YFV), and *Plasmodium* [109–111]. Microarray analyses indicate that *Wolbachia* induces the expression of Toll pathway and other immunity-related genes in stably trans-infected *Ae. aegypti* [70, 112, 113]. However, as *Wolbachia* restricts DENV in *Drosophila* and *Ae. albopictus* (two species with a long natural history of *Wolbachia* infection) in the absence of immune activation, it has been suggested that immune priming is not the fundamental mechanism of virus restriction, although it may enhance the trait in heterologous mosquito hosts [113, 114]. *Wolbachia* has also been shown to compete with the virus for crucial host resources [115], and to modulate the expression of certain mosquito microRNAs, thereby altering host gene expression to facilitate its own replication [116, 117].

In mosquitoes, *Wolbachia* is particularly suited for use in a population-replacement transmission-blocking strategy because of its ability to induce cytoplasmic incompatibility (CI), a phenomenon (maintained in stably trans-infected *Ae. aegypti*) in which crosses between uninfected females and infected males result in embryonic lethality (reviewed in [118]). This increases the reproductive success of infected females and allows *Wolbachia* to spread rapidly through insect populations despite possible fitness costs.

Sequencing-based, culture-independent approaches are increasingly being used to obtain comprehensive profiles of field mosquito microbiomes [119–122]. In *Anopheles gambiae*, the major African vector of malaria, targeted deep sequencing of microbial 16S ribosomal RNA revealed distinct gut microbiome communities at the aquatic larval and pupal stages and the terrestrial adult stage [119]. This finding is consistent with the fact that gut contents are usually cleared upon metamorphosis during the larvae-to-pupae and pupae-to-adult transitions [123], and implies that repopulation of the microbiome occurs at each stage. Bloodmeals drastically reduced gut microbiome diversity and led to an expansion of members of the *Enterobacteriaceae* family. These bacteria possess antioxidant mechanisms that may allow them to cope with the oxidative and nitrosative stresses associated with bloodmeal catabolism, suggesting that they benefit the mosquito by helping to maintain gut redox homeostasis [119].

1A study characterizing the microbiomes of wild-caught *Aedes*, *Anopheles*, and *Culex* mosquitoes from Kenya found that the gut microbiome of an individual adult mosquito was typically dominated by one bacterial taxon, while also containing many other much less abundant taxa. Although different mosquito species shared remarkably similar gut bacteria, there was enormous variation within individuals of the same species [120].

The composition and dynamics of endogenous mosquito gut microbiota may affect natural rates of disease transmission, as well as the success of transmission-blocking strategies that involve the introduction of native or non-native bacterial species into mosquito populations. Recent studies, for example, suggest that vertical transmission of *Wolbachia* in *An. gambiae* (another non-naturally infected mosquito species) is inhibited by native *Asaia* [124, 125]. The development of improved 16S sequencing methods that allow species-level identification [126], as well as metagenomic sequencing approaches that yield information on microbial function in addition to identity [127, 128], should help us understand complex relationships between bacterial communities and their insect hosts.

## Virus interactions with the human host

### Transcriptome profiling of the human host

DENV probably infects a wide range of cell types in the human host. Mouse studies suggest that hepatocytes are perhaps the most important cells for replication [129], but most human studies have concentrated on monocytes, macrophages, and dendritic cells [130, 131]. Acute disease, occurring 3–8 days after viral transmission from the mosquito, typically begins with a 3–7-day febrile phase, accompanied by symptoms such as headache, myalgia, arthralgia, retro-orbital pain, and rash. While most patients subsequently recover without complications, some progress to severe disease at around the time of defervescence (abatement of fever; reviewed in [132]).

Longitudinal studies using DNA microarray expression profiling to track transcriptomic changes in the blood of DENV-infected patients have identified two distinct phases of gene expression during the febrile stage. In the early acute phase (day 0–1, day 0 being the day of fever onset), genes associated with innate immunity, interferon (IFN)- and cytokine-mediated signaling, chemotaxis, and complement pathway activity reach peak expression but their expression declines by day 3–4, mirroring viremia levels. This marks a shift to the late acute phase, which is characterized by the expression of genes associated with the cell cycle and DNA repair, which peaks at day 5–6 [133, 134].

These results are consistent with cross-sectional studies that have identified IFN, NF-kB, Toll-like receptor (TLR), retinoic acid-inducible gene-I-like receptor (RLR), complement, and ubiquitin–proteasome pathway-related genes as prominent features of the febrile-stage transcriptional signature [135–140]. A number of these host responses look to have either pro-inflammatory profiles that may lead to later disease pathology or antiviral activities (or both) [137, 141], and may represent promising novel drug targets. The first clinical trial of a therapy exploiting a host target to inhibit viral replication did not, however, show sufficient activity [142]. The antiviral innate immune response profile wanes rapidly, and by the defervescent stage, transcripts of genes that are involved in biosynthesis, metabolism, and the adaptive immune response are most prominent [135, 136, 139, 140]; these may be less easily used as therapeutic targets.

Hemorrhagic manifestations leading to DSS typically appear around defervescence (day 4–7 of illness), when the host immune response is well established and viremia is rapidly declining. This suggests that vascular permeability is mediated by the host inflammatory response rather than by the virus directly. The onset of shock appears to be associated with an attenuated immune response, with several studies reporting reduced transcript abundances of IFN-stimulated and other innate immunity-related genes in DSS compared with those in well-matched DF patients prior to [143, 144] and at the point of defervescence [135, 139]. Thus, the host responses that contribute to vascular permeability may occur well before the onset of DSS, with rapid early disease progression being an important determinant of severe outcome, probably reflecting an earlier and larger peak viral load and a consequent earlier and larger host response [135].

Prospective studies designed to capture these early events found that dengue patients who eventually progress to DHF/DSS display an early increased abundance of transcripts associated with activated neutrophils, including those encoding granulocyte enzymes, membrane-bound integrin receptors, and microbicidal peptides such as defensins [136, 145, 146]. Several of these proteins might compromise capillary integrity — the serine proteases ELA2 and CTSG, for example, are known to cleave vascular endothelial cadherin [147]. It has thus been proposed that high viral antigen loads and immune complex formation (as seen in secondary dengue) during early infection induce neutrophil activation and degranulation, which then contribute to the triggering of vascular permeability [136]. Intriguingly, the platelet drop observed in patients and associated with disease severity may not be linked with these vascular permeability changes, but may instead be an independent event resulting from the inhibition of platelet production by the early inflammatory response [129]. (While most studies cited here classified patients as having DF, DHF, or DSS, we note that the WHO in 2009 revised its guidelines so that patients are now classified as having 'dengue with or without warning signs' or 'severe dengue' [148].)

While a detailed discussion is outside the scope of this review, techniques such as mass spectrometry and immunoassays have also been used to study human host responses to DENV infection and to distinguish mild from severe dengue disease at the proteome level [149–152].

### Genetic associations

In addition to expression profiling, genome-wide association studies (GWAS) have also contributed to our understanding of the pathogenesis of severe dengue. Strong associations with increased susceptibility to DSS have been identified at two distinct loci: *MICB* (*MHC class I polypeptide-related sequence B*), located within the major histocompatibility complex (MHC) region on chromosome 6; and *PLCE1* (*Phospholipase C*, *epsilon 1*), located on chromosome 10 (Table 1) [153].

*MICB* encodes an inducible activating ligand for the NKG2D type II receptor on natural killer (NK) cells and CD8+ T cells. Binding of MICB to NKG2D activates antiviral functions such as cytotoxic granule release and cytokine production [154]; it is possible that dysfunctional NK or CD8+ T-cell activation during early infection results in the higher viral burdens associated with severe dengue [155, 156]. Interestingly, a separate GWAS detected an association between the closely related *MICA* gene and hepatitis C virus (HCV)-induced hepatocellular carcinoma [152], suggesting an important role for the MIC proteins in flaviviral pathogenesis.

Mutations in *PLCE1* are also associated with nephrotic syndrome [157, 158], a childhood kidney disorder in which dysfunction of the glomerular basement membrane impairs blood filtering function, leading to hypovolemia in severe cases. This aspect of nephrotic syndrome shares striking similarities with DSS, and has led to the discovery that proteinuria may be predictive of severe dengue [159]. *PLCE1* has also been associated with blood pressure [160], suggesting a role in the maintenance of normal vascular endothelial barrier function. Disturbances in this vascular integrity may be the cause of DSS, offering the potential for a novel therapeutic approach to prevent it. This process may also go some way to explaining the association of DSS with pediatric dengue, as children are intrinsically more prone to vascular leak [161].

## Implications and future challenges for clinical management and transmission control

### Clinical management of dengue

Dengue is a significant burden on healthcare systems. Without specific antivirals, the case management of high-risk dengue patients is entirely supportive, involving constant monitoring and timely fluid support to prevent hypovolemic shock [132]. Nevertheless, the diverse clinical spectrum of dengue disease, as well as its initial similarity to other viral febrile illnesses, presents a challenge in the early identification of this relatively small high-risk group (perhaps 5 % of cases), resulting in the frequent hospitalization of patients with uncomplicated dengue or the non-hospitalization of patients who would benefit from interventions. WHO guidelines [148] recommend the use of warning signs to identify high-risk patients, but these have potential to be overly sensitive [162–164] and they generally occur during, or just one day before, the development of severe illness (4–7 days post-fever onset), providing only a narrow window for clinical intervention [164, 165].

Transcriptomic profiling of patients at early time points has greatly increased our understanding of dengue pathogenesis, and has identified host-response biomarkers that are associated with subsequent development of warning signs and progression to severe disease [133, 134, 136, 140, 144, 166]. Prognostic models combining mRNA and protein biomarkers with clinical parameters (such as platelet count) have also been developed and tested in proof-of-concept studies [133, 166, 167]. These have potential to further refine clinical triage, and would be particularly useful in primary healthcare settings; evaluation in larger prospective studies is needed for them to be applied more widely.

### Vaccine and drug development

There remains a pressing need for effective vaccines and specific antivirals against dengue. The approval in December 2015 of Sanofi-Pasteur's tetravalent vaccine Dengvaxia (CYD-TDV) for use in Mexico in a select age group (9–45 years) is certainly an achievement, but is unlikely to be a single solution. Although CYD-TDV is well tolerated in the short term and substantially reduces dengue hospitalizations, it shows serotype-specific efficacy, with less protection against serotype 2, and also provides limited protection against primary infection [14, 15]. Third-year follow-up data also indicate that CYD-TDV is associated with increased hospitalization risk for dengue in children below 9 years of age, raising the possibility that waning antibody titers predispose this age group to infection and more serious clinical presentations [12, 13], and highlighting the need for vaccines to elicit potent and balanced antibody responses even in dengue-naive recipients. On the therapeutics front, the candidate antivirals celgosivir (a host α-glucosidase inhibitor) and balapiravir (a nucleoside analog) were not found to be effective in clinical trials, despite promising activity in in vitro and animal models [142, 168]. This failure may be due to the very small window of therapeutic opportunity for antivirals, suggesting that prophylactic approaches might be required. In addition, anti-inflammatory approaches using re-purposed therapies have also proven to be ineffective to date [169, 170], although this might be due to their targeting of inappropriate host responses [171].

Efforts to develop improved next-generation vaccine and antiviral candidates will benefit from structural and functional genomics studies in both virus and host [172–174], which may identify regions of the viral genome [51, 58, 60] or novel host–viral interactions [141, 175] as potential targets.

Viral sequencing may be used to evaluate the effect of antivirals and vaccines on DENV populations, and to monitor the emergence of resistant or immune escape mutants. For example, although balapiravir induces C > N mutations by inhibiting the incorporation of cytosine bases into RNA templates by viral NS5 [176], deep sequencing revealed no differences in the frequency of these mutations between viral populations from drug- and placebo-treated patient groups [51]. This may provide a molecular explanation for its lack of efficacy in clinical trials [168].

In another study, DENV populations from mice treated with UV-4B, a host α-glucosidase inhibitor [177] soon to enter clinical trials, harbored significantly more variants than those from vehicle-treated mice. They also showed high ratios of non-synonymous to synonymous variants in the glycosylated M and NS1 proteins, suggesting that the drug is driving positive selection in these regions of the genome. Despite this, no escape mutants emerged even after multiple rounds of virus replication; the authors suggest that this reflects the better stability of antiviral approaches that target host factors [52].

### Control strategies targeting the mosquito vector

Novel control strategies targeting the mosquito vector are being tested in natural settings. Field releases of *Ae. aegypti* carrying the wMel strain of *Wolbachia* successfully introduced the bacterium into Australian mosquito populations, where it has remained established to date [111, 178]. Ongoing releases in Vietnam, Indonesia, Brazil, and Colombia [179], where dengue is much more common than in Australia, should yield information on the impact of population replacement on disease transmission.

Strategies involving genetically modified mosquitoes are also under development. The most advanced of these, termed release of insects carrying a dominant lethal allele (RIDL), seeks to eliminate vector populations by releasing males carrying a transgene that renders their offspring non-viable. One such construct induces cellular toxicity specifically in the flight muscles of female pupae, resulting in adult females that are unable to fly [180]; another induces lethality at the late larval or pupal stage [181]. Trials of RIDL mosquito strains have been carried out in the Cayman Islands, Brazil, and Malaysia by the company Oxitec, with a 95 % population reduction reported at the Brazilian field site [182–185].

Mosquito transcriptomics studies have yielded a plethora of DENV-responsive genes; these are increasingly being functionally characterized, and some have been found to play pro- or antiviral roles in the vector [186–189]. Such studies can identify candidate molecules for use in experimental transmission-blocking strategies, such as the transgenic overexpression of immune pathway activators or antiviral effectors [190–192], and the paratransgenic engineering of bacterial or fungal members of the microbiome to express anti-pathogen molecules [193–195]. Recent reports of *Anopheles* species engineered with the CRISPR-Cas9 gene drive system so that they are refractory to *Plasmodium* infection [196, 197] suggest that population replacement strategies are technically feasible, but should be adopted with caution [198].

In practice, control strategies targeting the vector will probably be complicated by genetic and transcriptomic divergence in mosquito and virus strains, and by the influence of the native gut microbiota. A combination of functional genomics and extensive field testing will most probably be required to overcome these challenges.

## Conclusion

In microbiology, there is increasing appreciation that host genetics, host gene expression, host immune background, and pathogen genetics are interrelated and should not be studied in isolation. The impact of DENV on the human host, in terms of clinical phenotype and host response, is shaped by host genetics, prior immune exposure, and virus genetics; in mosquitoes (and possibly even in humans), the gut microbiota adds an additional layer of complexity. Reciprocally, immune selection pressures exerted by either host shape the genetic diversity of DENV populations, potentially impacting their virulence, immunogenicity, or transmissibility.

Genomics approaches have allowed us to interrogate host–pathogen interactions on an unprecedented scale. This provides opportunities for integrating information from different taxa to attain a comprehensive picture of DENV in human and mosquito hosts. For example, with more whole-genome virus sequences becoming available, it will be possible to correlate DENV polymorphisms with host genotypes and clinical phenotypes, with specific immune pressures such as antiviral use, or with different subsets of mosquito gut bacteria. Continued dissection of such interactions to reveal their molecular mechanisms will provide new and better targets for the development of vaccines and antivirals, as well as for transmission-blocking strategies targeting the vector.

## Abbreviations

AMP: Antimicrobial peptide
C: Capsid
Dcr2: Dicer-2
DENV: Dengue virus
DF: Dengue fever
DHF: Dengue hemorrhagic fever
dsRNA: Double-stranded RNA
DSS: Dengue shock syndrome
DVRF1: Dengue virus restriction factor 1
E: Envelope
GWAS: Genome-wide association studies
IFN: Interferon
JAK-STAT: Janus kinase/signal transducers and activators of transcription
MHC: Major histocompatibility complex
*MICB*: *MHC class I polypeptide-related sequence B*
NK: Natural killer
NS: Non-structural
NTD: Neglected tropical disease
PIAS: Protein inhibitor of activated STAT
*PLCE1*: *Phospholipase C*, *epsilon 1*
prM: Premembrane
RNAi: RNA interference
siRNA: Small interfering RNA
SNP: Single-nucleotide polymorphism
WHO: World Health Organization
